# Supplementary material for: End Sequence Analysis Toolkit (ESAT) expands the extractable information from single-cell RNA-seq data
Source: Genome Res. 2016 Oct;26(10):1397–410. doi: 10.1101/gr.207902.116 (PMC5052061; doi:10.1101/gr.207902.116)
Supplement: Supplemental Material [file supp_gr.207902.116_Supp_Materials.tar.gz › ESAT-esat_v0.1/doc/html/interfaceumms_1_1core_1_1annotation_1_1_annotation_collection_3_01_t_01extends_01_annotation_01_4-members.html]

ESAT: Member List


ESAT


- Main Page
- Related Pages
- Packages
- Classes
- Files

- Class List
- Class Index
- Class Hierarchy
- Class Members

All Classes Namespaces Files Functions Variables Enumerator Pages

- umms
- core
- annotation
- AnnotationCollection< T extends Annotation >

umms.core.annotation.AnnotationCollection< T extends Annotation > Member List

This is the complete list of members for umms.core.annotation.AnnotationCollection< T extends Annotation >, including all inherited members.

|  |  |  |
| --- | --- | --- |
| addFilter(Predicate< T > filter) | umms.core.annotation.AnnotationCollection< T extends Annotation > |  |
| addFilters(Collection< Predicate< T >> filters) | umms.core.annotation.AnnotationCollection< T extends Annotation > |  |
| collapse(Annotation region, boolean fullyContained) | umms.core.annotation.AnnotationCollection< T extends Annotation > |  |
| getAverageCoverage(Annotation region) | umms.core.annotation.AnnotationCollection< T extends Annotation > |  |
| getBasesCovered(Annotation region, boolean fullyContained) | umms.core.annotation.AnnotationCollection< T extends Annotation > |  |
| getBasesCovered(Annotation region) | umms.core.annotation.AnnotationCollection< T extends Annotation > |  |
| getCoordinateSpace() | umms.core.annotation.AnnotationCollection< T extends Annotation > |  |
| getCount(Annotation region, boolean fullyContained) | umms.core.annotation.AnnotationCollection< T extends Annotation > |  |
| getCount(Annotation region) | umms.core.annotation.AnnotationCollection< T extends Annotation > |  |
| getCount(AnnotationList<?extends Annotation > set) | umms.core.annotation.AnnotationCollection< T extends Annotation > |  |
| getCount(AnnotationList<?extends Annotation > set, boolean fullyContained) | umms.core.annotation.AnnotationCollection< T extends Annotation > |  |
| getCountExcludingRegion(Annotation region, Annotation excluded) | umms.core.annotation.AnnotationCollection< T extends Annotation > |  |
| getCountStrandedExcludingRegion(Annotation region, Annotation excluded) | umms.core.annotation.AnnotationCollection< T extends Annotation > |  |
| getGlobalCount() | umms.core.annotation.AnnotationCollection< T extends Annotation > |  |
| getOverlappingAnnotations(Annotation region) | umms.core.annotation.AnnotationCollection< T extends Annotation > |  |
| getOverlappingAnnotations(Annotation region, boolean fullyContained) | umms.core.annotation.AnnotationCollection< T extends Annotation > |  |
| getPermutedAnnotations(Annotation region) | umms.core.annotation.AnnotationCollection< T extends Annotation > |  |
| getRefSequenceLambda(String refname) | umms.core.annotation.AnnotationCollection< T extends Annotation > |  |
| getTotalCoverage(Annotation region) | umms.core.annotation.AnnotationCollection< T extends Annotation > |  |
| iterator() | umms.core.annotation.AnnotationCollection< T extends Annotation > |  |
| scan(int windowSize, int overlap) | umms.core.annotation.AnnotationCollection< T extends Annotation > |  |
| scan(int windowSize, int overlap, WindowProcessor< W > processor) | umms.core.annotation.AnnotationCollection< T extends Annotation > |  |
| scan(Annotation region, int windowSize, int overlap, WindowProcessor< W > processor) | umms.core.annotation.AnnotationCollection< T extends Annotation > |  |
| scan(Annotation region, int windowSize, int overlap) | umms.core.annotation.AnnotationCollection< T extends Annotation > |  |
| size() | umms.core.annotation.AnnotationCollection< T extends Annotation > |  |


---

Generated on Fri Aug 15 2014 15:11:08 for ESAT by  

 1.8.7
